# 6-(4-Bromo­phen­yl)-2-eth­oxy-4-(2,4,5-trimethoxy­phen­yl)nicotinonitrile^1^

**DOI:** 10.1107/S1600536810005210

**Published:** 2010-02-17

**Authors:** Suchada Chantrapromma, Hoong-Kun Fun, Mahesh Padaki, Thitipone Suwunwong, Arun M. Isloor

**Affiliations:** a Crystal Materials Research Unit, Department of Chemistry, Faculty of Science, Prince of Songkla University, Hat-Yai, Songkhla 90112, Thailand; b X-ray Crystallography Unit, School of Physics, Universiti Sains Malaysia, 11800 USM, Penang, Malaysia; c Department of Chemistry, National Institute of Technology-Karnataka, Surathkal, Mangalore 575 025, India

## Abstract

There are two mol­ecules in the asymmetric unit of the title compound, C_23_H_21_BrN_2_O_4_, which differ in the conformation of  their ethoxy residues, *i.e.* almost co-planar with the pyridine ring in one mol­ecule [C—O—C—C = −174.0 (2)°] but almost perpendicular in the other [C—O—C—C = 92.8 (3)°]. The dihedral angles between the central pyridine ring and the 4-bromo­phenyl and 2,4,5-trimethoxy­phenyl rings are 11.05 (12) and 63.78 (12)°, respectively, in one mol­ecule; the corres­ponding angles in the other mol­ecule are 30.38 (13) and 65.38 (13)°, respectively. In the crystal structure, pairs of mol­ecules are arranged in a face-to-face sandwich structure which further stacks along the *b* axis. The crystal packing features C—H⋯π inter­actions and Br⋯O [3.5417 (17) Å], Br⋯C [3.748 (3) Å], C⋯N [3.376 (4) Å] and C⋯O [3.351 (3)–3.409 (3) Å] contacts. Finally, π⋯π inter­actions [centroid⋯centroid distances = 3.6346 (19) and 3.6882 (19) Å] are observed.

## Related literature

For hydrogen-bond motifs, see: Bernstein *et al.* (1995 ▶). For the synthesis and applications of nicotinonitrile derivatives, see: Borgna *et al.* (1993 ▶); Goda *et al.* (2004 ▶). For related structures, see Chantrapromma *et al.* (2009 ▶, 2010 ▶). For the stability of the temperature controller used in the data collection, see: Cosier & Glazer (1986 ▶).
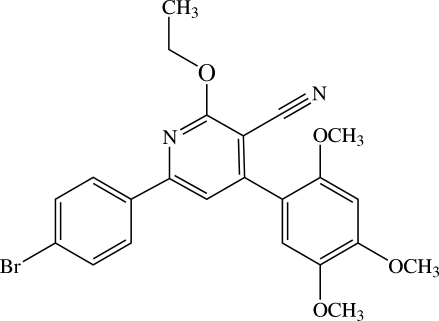


## Experimental

### 

#### Crystal data

C_23_H_21_BrN_2_O_4_
*M* *_r_* = 469.32
Triclinic, 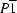

*a* = 7.9631 (2) Å
*b* = 11.0499 (3) Å
*c* = 23.9690 (6) Å
α = 92.201 (1)°
β = 91.968 (1)°
γ = 99.586 (1)°
*V* = 2076.31 (9) Å^3^
*Z* = 4
Mo *K*α radiation
μ = 2.01 mm^−1^
*T* = 100 K
0.59 × 0.10 × 0.05 mm

#### Data collection

Bruker APEXII CCD area-detector diffractometer
Absorption correction: multi-scan (*SADABS*; Bruker, 2005 ▶) *T*_min_ = 0.384, *T*_max_ = 0.899
31800 measured reflections
9488 independent reflections
7074 reflections with *I* > 2σ(*I*)
*R*_int_ = 0.043

#### Refinement

*R*[*F* ^2^ > 2σ(*F* ^2^)] = 0.036
*wR*(*F* ^2^) = 0.098
*S* = 1.07
9488 reflections
549 parameters
H-atom parameters constrained
Δρ_max_ = 0.79 e Å^−3^
Δρ_min_ = −0.38 e Å^−3^

### 

Data collection: *APEX2* (Bruker, 2005 ▶); cell refinement: *SAINT* (Bruker, 2005 ▶); data reduction: *SAINT*; program(s) used to solve structure: *SHELXTL* (Sheldrick, 2008 ▶); program(s) used to refine structure: *SHELXTL*; molecular graphics: *SHELXTL*; software used to prepare material for publication: *SHELXTL* and *PLATON* (Spek, 2009 ▶).

## Supplementary Material

Crystal structure: contains datablocks global, I. DOI: 10.1107/S1600536810005210/tk2622sup1.cif

Structure factors: contains datablocks I. DOI: 10.1107/S1600536810005210/tk2622Isup2.hkl

Additional supplementary materials:  crystallographic information; 3D view; checkCIF report

## Figures and Tables

**Table 1:** Hydrogen-bond geometry (Å, °) *Cg*1 and *Cg*2 are the centroids of C7*A*–C11*A*/N1*A* and C12*A*–C17*A* rings, respectively.

*D*—H⋯*A*	*D*—H	H⋯*A*	*D*⋯*A*	*D*—H⋯*A*
C22*A*—H22*C*⋯*Cg*2^i^	0.96	2.74	3.585 (3)	147
C22*B*—H22*E*⋯*Cg*1^ii^	0.96	2.68	3.511 (3)	145

Symmetry codes: (i) 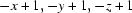; (ii) 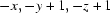.

**Fig. 1.**
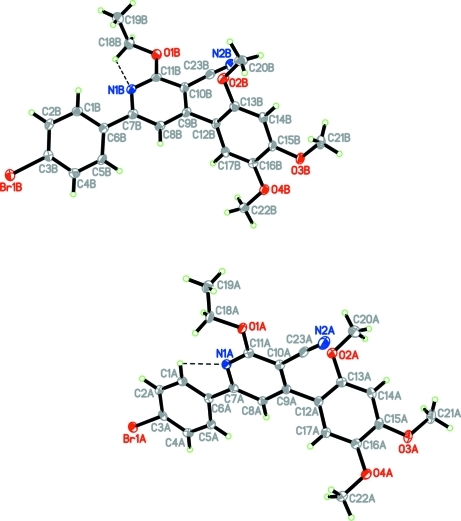
The molecular structure of the title compound, showing 50% probability displacement ellipsoids and the atom-numbering scheme. Intramolecular C—H···N interactions are shown as dashed lines.

**Fig. 2.**
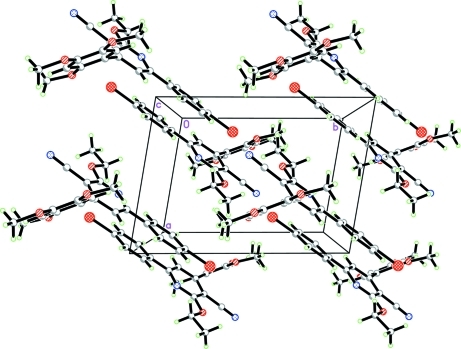
The crystal packing of the title compound viewed along the c axis.

## supplementary crystallographic information

### Comment

Substituted pyridine derivatives have been claimed to have several biological activities (Borgna *et al.*, 1993; Goda *et al.*, 2004). We have previously reported the syntheses and crystal structures of the nicotinonitrile derivatives (Chantrapromma *et al.*, 2009; 2010). In continuation of our research into the synthesis of antimicrobial agents, the title malononitrile derivative was synthesised and studied for its anti-bacterial activities. However, our results showed that the title compound does not possess anti-bacterial activities. Herein, we report the crystal structure of the title compound (I).

There are two crystallographic independent molecules *A* and *B* in the asymmetric unit of (I) (Fig. 1) with differences in conformation of the ethoxy group distinguishing them. In molecule *A* the ethoxy residue is almost co-planar with the pyridine ring [C11A–O1A–C18A–C19A = -174.0 (2) °] whereas it is almost perpendicular in molecule *B* [C11B–O1B–C18B–C19B = 92.8 (3) °]. The dihedral angles between the central pyridine ring and the 4-bromophenyl and 2,4,5-trimethoxyphenyl rings are 11.05 (12) and 63.78 (12) ° respectively in molecule *A* whereas the corresponding pair of angles in molecule *B* are 30.38 (13) and 65.38 (13) °. All three methoxy groups are nearly co-planar with the attached benzene ring [torsion angles C20–O2–C13–C14 = -11.6 (4) °, C21–O3–C15–C16 = 180.0 (2) ° and C22–O4–C16–C17 = 8.1 (4) ° in molecule *A*; and the corresponding values are -5.5 (4), -175.5 (2) and -7.7 (4) ° in molecule *B*]. Weak intramolecular C1A—H1AA···N1A (in molecule *A*) and C18B–H18C—N1B (in molecule *B*) interactions generate S(5) ring motifs (Bernstein *et al.*, 1995). The bond distances are comparable with those in closely related structures (Chantrapromma *et al.*, 2009; 2010).

In the crystal structure (Fig. 2), the molecules are arranged into a face-to-face sandwich-like structure which further stack along the *b* axis. The crystal is consolidated by C—H···π interactions (Table 1) and Br···O [3.5417 (17) Å], Br···C [3.748 (3) Å], C···N [3.376 (4) Å] and C···O [3.351 (3) - 3.409 (3) Å] contacts. Finally, π···π interactions with the distances of Cg_1_···Cg_4_ = 3.6346 (15) Å (symmetry code: 1-x, 1-y, 1-z) and Cg_2_···Cg_3_ = 3.6882 (15) Å (symmetry code: -x, 1-y, 1-z) are observed; Cg_1_, Cg_2_, Cg_3_ and Cg_4_ are the centroids of C7A–C11A/N1A, C12A–C17A, C7B–C11B/N1B, and C12B–C17B rings, respectively.

### Experimental

(*E*)-1-(4-Bromophenyl)-3-(2,4,5-trimethoxyphenyl)prop-2-en-1-one (0.57 g, 0.0015 mol) was added with continuous stirring to a freshly prepared sodium alkoxide solution (0.0014 mol of sodium in 100 ml of ethanol). Malononitrile (1.3 g, 0.02 mol) was then added with continuous stirring at room temperature until the precipitate separated out. The resulting solid was filtered (yield 68 %). Colorless needle-shaped single crystals of the title compound were obtained by recrystallization from ethanol by the slow evaporation of the solvent at room temperature after several days, Mp. 460-461 K.

### Refinement

All H atoms were positioned geometrically and allowed to ride on their parent atoms, with d(C—H) = 0.93 Å for aromatic-H, 0.97 for CH_2_ and 0.96 Å for methyl-H atoms. The *U*_iso_ values were constrained to be 1.5*U*_eq_ of the carrier atom for methyl-H atoms and 1.2*U*_eq_ for the remaining H atoms. A rotating group model was used for the methyl groups.

### Crystal data

**Table:**

C_23_H_21_BrN_2_O_4_	*Z* = 4
*M**_r_* = 469.32	*F*(000) = 960
Triclinic, *P*1	*D*_x_ = 1.501 Mg m^−^^3^
Hall symbol: -P 1	Melting point = 460–461 K
*a* = 7.9631 (2) Å	Mo *K*α radiation, λ = 0.71073 Å
*b* = 11.0499 (3) Å	Cell parameters from 9488 reflections
*c* = 23.9690 (6) Å	θ = 0.9–27.5°
α = 92.201 (1)°	µ = 2.01 mm^−^^1^
β = 91.968 (1)°	*T* = 100 K
γ = 99.586 (1)°	Needle, colorless
*V* = 2076.31 (9) Å^3^	0.59 × 0.10 × 0.05 mm

### Data collection

**Table:**

Bruker APEXII CCD area-detector diffractometer	9488 independent reflections
Radiation source: sealed tube	7074 reflections with *I* > 2σ(*I*)
graphite	*R*_int_ = 0.043
φ and ω scans	θ_max_ = 27.5°, θ_min_ = 0.9°
Absorption correction: multi-scan (*SADABS*; Bruker, 2005)	*h* = −10→10
*T*_min_ = 0.384, *T*_max_ = 0.899	*k* = −14→14
31800 measured reflections	*l* = −31→31

### Refinement

**Table:**

Refinement on *F*^2^	Primary atom site location: structure-invariant direct methods
Least-squares matrix: full	Secondary atom site location: difference Fourier map
*R*[*F*^2^ > 2σ(*F*^2^)] = 0.036	Hydrogen site location: inferred from neighbouring sites
*wR*(*F*^2^) = 0.098	H-atom parameters constrained
*S* = 1.07	*w* = 1/[σ^2^(*F*_o_^2^) + (0.0443*P*)^2^ + 0.7288*P*] where *P* = (*F*_o_^2^ + 2*F*_c_^2^)/3
9488 reflections	(Δ/σ)_max_ = 0.001
549 parameters	Δρ_max_ = 0.79 e Å^−^^3^
0 restraints	Δρ_min_ = −0.38 e Å^−^^3^

### Special details

**Table:**

Experimental. The crystal was placed in the cold stream of an Oxford Cryosystems Cobra open-flow nitrogen cryostat (Cosier & Glazer, 1986) operating at 100.0 (1) K.
Geometry. All esds (except the esd in the dihedral angle between two l.s. planes) are estimated using the full covariance matrix. The cell esds are taken into account individually in the estimation of esds in distances, angles and torsion angles; correlations between esds in cell parameters are only used when they are defined by crystal symmetry. An approximate (isotropic) treatment of cell esds is used for estimating esds involving l.s. planes.
Refinement. Refinement of *F*^2^ against ALL reflections. The weighted *R*-factor wR and goodness of fit *S* are based on *F*^2^, conventional *R*-factors *R* are based on *F*, with *F* set to zero for negative *F*^2^. The threshold expression of *F*^2^ > σ(*F*^2^) is used only for calculating *R*-factors(gt) etc. and is not relevant to the choice of reflections for refinement. *R*-factors based on *F*^2^ are statistically about twice as large as those based on *F*, and *R*- factors based on ALL data will be even larger.

### Fractional atomic coordinates and isotropic or equivalent isotropic displacement parameters (Å^2^)

**Table:**

	*x*	*y*	*z*	*U*_iso_*/*U*_eq_	
Br1A	−0.17700 (4)	−0.35388 (2)	0.439858 (11)	0.02094 (8)	
O1A	0.5666 (2)	0.35133 (16)	0.42626 (7)	0.0190 (4)	
O2A	0.2036 (3)	0.48545 (17)	0.28313 (7)	0.0229 (4)	
O3A	0.2540 (3)	0.46349 (17)	0.08142 (7)	0.0227 (4)	
O4A	0.3749 (3)	0.26013 (17)	0.08836 (7)	0.0211 (4)	
N1A	0.3634 (3)	0.18217 (19)	0.40057 (8)	0.0161 (5)	
N2A	0.6447 (4)	0.5362 (2)	0.30967 (10)	0.0304 (6)	
C1A	0.1472 (3)	−0.0213 (2)	0.43550 (10)	0.0171 (6)	
H1AA	0.2118	0.0341	0.4613	0.021*	
C2A	0.0518 (4)	−0.1272 (2)	0.45413 (11)	0.0183 (6)	
H2AA	0.0519	−0.1434	0.4919	0.022*	
C3A	−0.0433 (3)	−0.2081 (2)	0.41548 (11)	0.0160 (5)	
C4A	−0.0472 (3)	−0.1856 (2)	0.35901 (11)	0.0177 (6)	
H4AA	−0.1134	−0.2411	0.3337	0.021*	
C5A	0.0488 (4)	−0.0796 (2)	0.34091 (10)	0.0179 (6)	
H5AA	0.0470	−0.0639	0.3031	0.022*	
C6A	0.1488 (3)	0.0047 (2)	0.37885 (10)	0.0145 (5)	
C7A	0.2528 (3)	0.1189 (2)	0.36092 (10)	0.0153 (5)	
C8A	0.2379 (3)	0.1613 (2)	0.30754 (10)	0.0157 (5)	
H8AA	0.1637	0.1154	0.2809	0.019*	
C9A	0.3339 (4)	0.2727 (2)	0.29383 (10)	0.0163 (5)	
C10A	0.4480 (3)	0.3366 (2)	0.33414 (10)	0.0160 (5)	
C11A	0.4564 (3)	0.2863 (2)	0.38748 (10)	0.0158 (5)	
C12A	0.3084 (3)	0.3240 (2)	0.23801 (10)	0.0166 (6)	
C13A	0.2479 (3)	0.4339 (2)	0.23373 (11)	0.0183 (6)	
C14A	0.2272 (3)	0.4832 (2)	0.18187 (11)	0.0186 (6)	
H14A	0.1854	0.5566	0.1793	0.022*	
C15A	0.2695 (3)	0.4223 (2)	0.13403 (10)	0.0180 (6)	
C16A	0.3318 (3)	0.3113 (2)	0.13770 (10)	0.0180 (6)	
C17A	0.3479 (3)	0.2623 (2)	0.18950 (10)	0.0166 (5)	
H17A	0.3856	0.1873	0.1920	0.020*	
C18A	0.5755 (4)	0.2961 (2)	0.48023 (10)	0.0206 (6)	
H18A	0.6219	0.2205	0.4764	0.025*	
H18B	0.4625	0.2770	0.4949	0.025*	
C19A	0.6888 (4)	0.3869 (3)	0.51911 (11)	0.0244 (6)	
H19A	0.6960	0.3531	0.5552	0.037*	
H19B	0.6423	0.4615	0.5224	0.037*	
H19C	0.8007	0.4042	0.5045	0.037*	
C20A	0.1635 (5)	0.6051 (3)	0.28312 (13)	0.0425 (9)	
H20A	0.1407	0.6307	0.3204	0.064*	
H20B	0.0645	0.6053	0.2591	0.064*	
H20C	0.2579	0.6607	0.2698	0.064*	
C21A	0.1906 (4)	0.5766 (3)	0.07668 (12)	0.0281 (7)	
H21A	0.1801	0.5945	0.0380	0.042*	
H21B	0.2683	0.6418	0.0960	0.042*	
H21C	0.0810	0.5695	0.0929	0.042*	
C22A	0.4189 (4)	0.1406 (2)	0.09113 (11)	0.0243 (6)	
H22A	0.4428	0.1117	0.0544	0.036*	
H22B	0.3256	0.0854	0.1053	0.036*	
H22C	0.5179	0.1440	0.1155	0.036*	
C23A	0.5564 (4)	0.4488 (3)	0.32183 (10)	0.0215 (6)	
Br1B	0.38606 (4)	0.11796 (3)	0.926074 (11)	0.02560 (9)	
O1B	0.0191 (3)	0.84937 (17)	0.92736 (7)	0.0231 (4)	
O2B	0.4260 (3)	0.98663 (17)	0.78188 (7)	0.0240 (4)	
O3B	0.2913 (3)	0.96201 (16)	0.58180 (7)	0.0210 (4)	
O4B	0.0815 (3)	0.75855 (16)	0.59168 (7)	0.0209 (4)	
N1B	0.1491 (3)	0.68209 (19)	0.90306 (8)	0.0164 (5)	
N2B	−0.0051 (3)	1.0323 (2)	0.81419 (10)	0.0279 (6)	
C1B	0.3117 (4)	0.4800 (2)	0.93221 (11)	0.0199 (6)	
H1BA	0.3184	0.5436	0.9592	0.024*	
C2B	0.3487 (4)	0.3679 (2)	0.94771 (11)	0.0202 (6)	
H2BA	0.3800	0.3556	0.9845	0.024*	
C3B	0.3378 (4)	0.2743 (2)	0.90675 (11)	0.0199 (6)	
C4B	0.2924 (4)	0.2911 (3)	0.85171 (11)	0.0230 (6)	
H4BA	0.2850	0.2270	0.8250	0.028*	
C5B	0.2584 (4)	0.4043 (2)	0.83709 (11)	0.0226 (6)	
H5BA	0.2310	0.4170	0.8000	0.027*	
C6B	0.2645 (3)	0.5004 (2)	0.87740 (10)	0.0173 (6)	
C7B	0.2157 (3)	0.6191 (2)	0.86213 (11)	0.0178 (6)	
C8B	0.2394 (4)	0.6627 (2)	0.80881 (10)	0.0187 (6)	
H8BA	0.2854	0.6167	0.7818	0.022*	
C9B	0.1946 (3)	0.7745 (2)	0.79583 (10)	0.0165 (5)	
C10B	0.1183 (3)	0.8366 (2)	0.83705 (10)	0.0179 (6)	
C11B	0.0976 (4)	0.7856 (2)	0.88999 (10)	0.0179 (6)	
C12B	0.2257 (3)	0.8253 (2)	0.74001 (10)	0.0164 (5)	
C13B	0.3413 (4)	0.9333 (2)	0.73404 (11)	0.0185 (6)	
C14B	0.3669 (4)	0.9808 (2)	0.68134 (10)	0.0178 (6)	
H14B	0.4443	1.0526	0.6774	0.021*	
C15B	0.2768 (4)	0.9209 (2)	0.63469 (10)	0.0170 (6)	
C16B	0.1637 (3)	0.8103 (2)	0.64004 (10)	0.0160 (5)	
C17B	0.1417 (3)	0.7636 (2)	0.69231 (10)	0.0172 (6)	
H17B	0.0693	0.6893	0.6959	0.021*	
C18B	−0.0121 (4)	0.8016 (3)	0.98201 (11)	0.0262 (7)	
H18C	−0.0237	0.7127	0.9796	0.031*	
H18D	−0.1179	0.8227	0.9951	0.031*	
C19B	0.1320 (5)	0.8537 (3)	1.02274 (13)	0.0367 (8)	
H19D	0.1053	0.8262	1.0594	0.055*	
H19E	0.1481	0.9418	1.0233	0.055*	
H19F	0.2346	0.8265	1.0115	0.055*	
C20B	0.5570 (4)	1.0900 (3)	0.77626 (12)	0.0278 (7)	
H20D	0.6111	1.1153	0.8122	0.042*	
H20E	0.5081	1.1564	0.7614	0.042*	
H20F	0.6400	1.0679	0.7514	0.042*	
C21B	0.4143 (4)	1.0690 (3)	0.57435 (11)	0.0233 (6)	
H21D	0.4166	1.0868	0.5355	0.035*	
H21E	0.5248	1.0550	0.5871	0.035*	
H21F	0.3845	1.1373	0.5955	0.035*	
C22B	−0.0187 (4)	0.6395 (2)	0.59614 (11)	0.0227 (6)	
H22D	−0.0687	0.6101	0.5601	0.034*	
H22E	−0.1074	0.6447	0.6218	0.034*	
H22F	0.0526	0.5839	0.6095	0.034*	
C23B	0.0514 (4)	0.9469 (3)	0.82524 (11)	0.0212 (6)	

### Atomic displacement parameters (Å^2^)

**Table:**

	*U*^11^	*U*^22^	*U*^33^	*U*^12^	*U*^13^	*U*^23^
Br1A	0.02292 (16)	0.01573 (13)	0.02271 (14)	−0.00184 (12)	0.00184 (12)	0.00381 (10)
O1A	0.0229 (11)	0.0180 (9)	0.0136 (8)	−0.0040 (8)	−0.0027 (8)	0.0030 (7)
O2A	0.0305 (12)	0.0210 (10)	0.0184 (9)	0.0076 (9)	0.0038 (9)	0.0017 (8)
O3A	0.0238 (11)	0.0271 (10)	0.0185 (9)	0.0066 (9)	0.0006 (8)	0.0092 (8)
O4A	0.0245 (11)	0.0247 (10)	0.0153 (9)	0.0070 (9)	0.0012 (8)	0.0012 (7)
N1A	0.0153 (12)	0.0162 (11)	0.0161 (10)	0.0003 (10)	0.0009 (9)	0.0014 (8)
N2A	0.0366 (16)	0.0297 (14)	0.0200 (12)	−0.0087 (13)	−0.0015 (12)	0.0031 (10)
C1A	0.0157 (14)	0.0176 (13)	0.0175 (12)	0.0016 (11)	−0.0003 (11)	−0.0007 (10)
C2A	0.0217 (15)	0.0186 (13)	0.0148 (12)	0.0041 (12)	0.0008 (11)	0.0024 (10)
C3A	0.0123 (13)	0.0121 (12)	0.0238 (13)	0.0014 (11)	0.0036 (11)	0.0034 (10)
C4A	0.0135 (14)	0.0179 (13)	0.0209 (13)	0.0015 (11)	−0.0005 (11)	−0.0030 (10)
C5A	0.0205 (15)	0.0185 (13)	0.0144 (12)	0.0022 (12)	0.0001 (11)	0.0003 (10)
C6A	0.0080 (13)	0.0162 (12)	0.0194 (12)	0.0023 (11)	0.0006 (11)	0.0022 (10)
C7A	0.0127 (13)	0.0165 (12)	0.0175 (12)	0.0046 (11)	0.0026 (11)	−0.0007 (10)
C8A	0.0135 (14)	0.0171 (12)	0.0159 (12)	0.0018 (11)	−0.0023 (11)	−0.0012 (10)
C9A	0.0173 (14)	0.0178 (13)	0.0148 (12)	0.0050 (11)	0.0024 (11)	0.0010 (10)
C10A	0.0112 (13)	0.0176 (13)	0.0187 (12)	0.0006 (11)	0.0009 (11)	0.0012 (10)
C11A	0.0114 (13)	0.0189 (13)	0.0170 (12)	0.0030 (11)	−0.0006 (11)	−0.0006 (10)
C12A	0.0163 (14)	0.0165 (12)	0.0156 (12)	−0.0017 (11)	−0.0009 (11)	0.0023 (10)
C13A	0.0146 (14)	0.0190 (13)	0.0200 (13)	0.0000 (12)	0.0011 (11)	−0.0011 (10)
C14A	0.0165 (14)	0.0165 (13)	0.0232 (13)	0.0031 (12)	0.0014 (12)	0.0040 (10)
C15A	0.0151 (14)	0.0206 (13)	0.0169 (12)	−0.0026 (12)	0.0003 (11)	0.0054 (10)
C16A	0.0143 (14)	0.0199 (13)	0.0184 (13)	−0.0009 (12)	0.0012 (11)	0.0001 (10)
C17A	0.0131 (14)	0.0170 (12)	0.0183 (12)	−0.0012 (11)	−0.0010 (11)	0.0011 (10)
C18A	0.0254 (16)	0.0215 (13)	0.0142 (12)	0.0011 (13)	−0.0005 (12)	0.0065 (10)
C19A	0.0235 (16)	0.0273 (15)	0.0206 (14)	−0.0011 (13)	−0.0028 (12)	0.0032 (11)
C20A	0.071 (3)	0.0301 (17)	0.0321 (17)	0.0255 (19)	0.0053 (18)	0.0008 (14)
C21A	0.0297 (18)	0.0338 (16)	0.0237 (14)	0.0107 (15)	0.0022 (13)	0.0138 (12)
C22A	0.0315 (18)	0.0239 (14)	0.0173 (13)	0.0054 (14)	−0.0009 (13)	−0.0013 (11)
C23A	0.0262 (16)	0.0238 (14)	0.0126 (12)	−0.0010 (13)	−0.0007 (12)	0.0013 (11)
Br1B	0.03322 (19)	0.02264 (15)	0.02261 (14)	0.00896 (13)	0.00077 (13)	0.00399 (11)
O1B	0.0277 (12)	0.0230 (10)	0.0192 (9)	0.0039 (9)	0.0058 (9)	0.0030 (8)
O2B	0.0216 (11)	0.0272 (10)	0.0189 (9)	−0.0078 (9)	−0.0012 (8)	0.0018 (8)
O3B	0.0241 (11)	0.0224 (10)	0.0155 (9)	−0.0003 (9)	0.0016 (8)	0.0065 (7)
O4B	0.0233 (11)	0.0212 (9)	0.0160 (9)	−0.0030 (9)	−0.0007 (8)	0.0027 (7)
N1B	0.0122 (11)	0.0194 (11)	0.0160 (10)	−0.0021 (10)	−0.0012 (9)	0.0011 (9)
N2B	0.0287 (15)	0.0261 (13)	0.0282 (13)	0.0037 (12)	−0.0045 (11)	0.0023 (10)
C1B	0.0195 (15)	0.0201 (13)	0.0186 (13)	−0.0010 (12)	0.0011 (12)	−0.0008 (10)
C2B	0.0182 (15)	0.0256 (14)	0.0164 (12)	0.0018 (12)	−0.0012 (12)	0.0048 (11)
C3B	0.0167 (15)	0.0202 (13)	0.0234 (14)	0.0036 (12)	0.0023 (12)	0.0050 (11)
C4B	0.0270 (17)	0.0222 (14)	0.0199 (13)	0.0047 (13)	0.0020 (12)	−0.0021 (11)
C5B	0.0273 (16)	0.0260 (14)	0.0144 (12)	0.0039 (13)	−0.0003 (12)	0.0038 (11)
C6B	0.0147 (14)	0.0196 (13)	0.0163 (12)	−0.0016 (11)	0.0016 (11)	0.0018 (10)
C7B	0.0121 (14)	0.0210 (13)	0.0187 (13)	−0.0020 (11)	−0.0019 (11)	0.0018 (10)
C8B	0.0164 (14)	0.0224 (14)	0.0166 (12)	0.0014 (12)	0.0019 (11)	0.0003 (10)
C9B	0.0108 (13)	0.0213 (13)	0.0149 (12)	−0.0041 (11)	−0.0025 (10)	0.0033 (10)
C10B	0.0138 (14)	0.0205 (13)	0.0179 (12)	−0.0015 (12)	−0.0023 (11)	0.0032 (10)
C11B	0.0170 (14)	0.0181 (13)	0.0160 (12)	−0.0037 (12)	−0.0008 (11)	−0.0013 (10)
C12B	0.0141 (14)	0.0181 (13)	0.0172 (12)	0.0027 (11)	0.0007 (11)	0.0038 (10)
C13B	0.0163 (14)	0.0203 (13)	0.0186 (13)	0.0025 (12)	−0.0009 (11)	0.0015 (10)
C14B	0.0159 (14)	0.0165 (13)	0.0210 (13)	0.0019 (11)	0.0024 (11)	0.0040 (10)
C15B	0.0186 (15)	0.0186 (13)	0.0156 (12)	0.0063 (12)	0.0038 (11)	0.0051 (10)
C16B	0.0099 (13)	0.0196 (13)	0.0191 (13)	0.0041 (11)	0.0012 (11)	−0.0001 (10)
C17B	0.0148 (14)	0.0164 (12)	0.0205 (13)	0.0012 (11)	0.0035 (11)	0.0049 (10)
C18B	0.0331 (18)	0.0266 (15)	0.0195 (13)	0.0043 (14)	0.0077 (13)	0.0036 (11)
C19B	0.049 (2)	0.0237 (15)	0.0343 (17)	−0.0022 (16)	−0.0071 (16)	0.0035 (13)
C20B	0.0288 (18)	0.0264 (15)	0.0237 (14)	−0.0074 (14)	0.0019 (13)	−0.0040 (12)
C21B	0.0173 (15)	0.0282 (15)	0.0238 (14)	−0.0005 (13)	0.0019 (12)	0.0110 (12)
C22B	0.0263 (16)	0.0195 (13)	0.0205 (13)	−0.0015 (13)	0.0006 (12)	0.0001 (11)
C23B	0.0206 (15)	0.0254 (15)	0.0153 (13)	−0.0024 (13)	−0.0013 (12)	0.0012 (11)

### Geometric parameters (Å, °)

**Table:**

Br1A—C3A	1.901 (2)	Br1B—C3B	1.902 (3)
O1A—C11A	1.354 (3)	O1B—C11B	1.352 (3)
O1A—C18A	1.456 (3)	O1B—C18B	1.445 (3)
O2A—C13A	1.377 (3)	O2B—C13B	1.371 (3)
O2A—C20A	1.411 (3)	O2B—C20B	1.427 (3)
O3A—C15A	1.366 (3)	O3B—C15B	1.365 (3)
O3A—C21A	1.431 (3)	O3B—C21B	1.425 (3)
O4A—C16A	1.370 (3)	O4B—C16B	1.368 (3)
O4A—C22A	1.426 (3)	O4B—C22B	1.430 (3)
N1A—C11A	1.316 (3)	N1B—C11B	1.322 (3)
N1A—C7A	1.362 (3)	N1B—C7B	1.354 (3)
N2A—C23A	1.150 (3)	N2B—C23B	1.147 (4)
C1A—C2A	1.384 (4)	C1B—C2B	1.381 (4)
C1A—C6A	1.398 (3)	C1B—C6B	1.391 (3)
C1A—H1AA	0.9300	C1B—H1BA	0.9300
C2A—C3A	1.376 (4)	C2B—C3B	1.388 (4)
C2A—H2AA	0.9300	C2B—H2BA	0.9300
C3A—C4A	1.386 (4)	C3B—C4B	1.383 (4)
C4A—C5A	1.381 (4)	C4B—C5B	1.379 (4)
C4A—H4AA	0.9300	C4B—H4BA	0.9300
C5A—C6A	1.402 (3)	C5B—C6B	1.402 (4)
C5A—H5AA	0.9300	C5B—H5BA	0.9300
C6A—C7A	1.477 (4)	C6B—C7B	1.485 (4)
C7A—C8A	1.387 (3)	C7B—C8B	1.391 (3)
C8A—C9A	1.393 (4)	C8B—C9B	1.385 (4)
C8A—H8AA	0.9300	C8B—H8BA	0.9300
C9A—C10A	1.390 (4)	C9B—C10B	1.394 (4)
C9A—C12A	1.494 (3)	C9B—C12B	1.484 (3)
C10A—C11A	1.417 (3)	C10B—C11B	1.412 (3)
C10A—C23A	1.435 (4)	C10B—C23B	1.443 (4)
C12A—C13A	1.384 (4)	C12B—C13B	1.396 (4)
C12A—C17A	1.398 (4)	C12B—C17B	1.399 (4)
C13A—C14A	1.392 (4)	C13B—C14B	1.395 (3)
C14A—C15A	1.387 (4)	C14B—C15B	1.390 (4)
C14A—H14A	0.9300	C14B—H14B	0.9300
C15A—C16A	1.402 (4)	C15B—C16B	1.406 (4)
C16A—C17A	1.384 (3)	C16B—C17B	1.379 (3)
C17A—H17A	0.9300	C17B—H17B	0.9300
C18A—C19A	1.503 (4)	C18B—C19B	1.504 (4)
C18A—H18A	0.9700	C18B—H18C	0.9700
C18A—H18B	0.9700	C18B—H18D	0.9700
C19A—H19A	0.9600	C19B—H19D	0.9600
C19A—H19B	0.9600	C19B—H19E	0.9600
C19A—H19C	0.9600	C19B—H19F	0.9600
C20A—H20A	0.9600	C20B—H20D	0.9600
C20A—H20B	0.9600	C20B—H20E	0.9600
C20A—H20C	0.9600	C20B—H20F	0.9600
C21A—H21A	0.9600	C21B—H21D	0.9600
C21A—H21B	0.9600	C21B—H21E	0.9600
C21A—H21C	0.9600	C21B—H21F	0.9600
C22A—H22A	0.9600	C22B—H22D	0.9600
C22A—H22B	0.9600	C22B—H22E	0.9600
C22A—H22C	0.9600	C22B—H22F	0.9600
			
C11A—O1A—C18A	115.60 (19)	C11B—O1B—C18B	119.1 (2)
C13A—O2A—C20A	118.5 (2)	C13B—O2B—C20B	117.6 (2)
C15A—O3A—C21A	116.7 (2)	C15B—O3B—C21B	117.0 (2)
C16A—O4A—C22A	115.9 (2)	C16B—O4B—C22B	115.60 (19)
C11A—N1A—C7A	118.5 (2)	C11B—N1B—C7B	117.5 (2)
C2A—C1A—C6A	121.6 (2)	C2B—C1B—C6B	121.9 (2)
C2A—C1A—H1AA	119.2	C2B—C1B—H1BA	119.1
C6A—C1A—H1AA	119.2	C6B—C1B—H1BA	119.1
C3A—C2A—C1A	118.5 (2)	C1B—C2B—C3B	118.1 (2)
C3A—C2A—H2AA	120.8	C1B—C2B—H2BA	121.0
C1A—C2A—H2AA	120.8	C3B—C2B—H2BA	121.0
C2A—C3A—C4A	121.9 (2)	C4B—C3B—C2B	121.9 (2)
C2A—C3A—Br1A	119.47 (19)	C4B—C3B—Br1B	118.4 (2)
C4A—C3A—Br1A	118.59 (19)	C2B—C3B—Br1B	119.7 (2)
C5A—C4A—C3A	119.0 (2)	C5B—C4B—C3B	119.0 (2)
C5A—C4A—H4AA	120.5	C5B—C4B—H4BA	120.5
C3A—C4A—H4AA	120.5	C3B—C4B—H4BA	120.5
C4A—C5A—C6A	120.9 (2)	C4B—C5B—C6B	120.9 (2)
C4A—C5A—H5AA	119.6	C4B—C5B—H5BA	119.6
C6A—C5A—H5AA	119.6	C6B—C5B—H5BA	119.6
C1A—C6A—C5A	118.1 (2)	C1B—C6B—C5B	118.3 (2)
C1A—C6A—C7A	119.7 (2)	C1B—C6B—C7B	121.1 (2)
C5A—C6A—C7A	122.3 (2)	C5B—C6B—C7B	120.6 (2)
N1A—C7A—C8A	121.5 (2)	N1B—C7B—C8B	122.5 (2)
N1A—C7A—C6A	115.7 (2)	N1B—C7B—C6B	116.2 (2)
C8A—C7A—C6A	122.7 (2)	C8B—C7B—C6B	121.3 (2)
C7A—C8A—C9A	120.1 (2)	C9B—C8B—C7B	120.1 (2)
C7A—C8A—H8AA	119.9	C9B—C8B—H8BA	119.9
C9A—C8A—H8AA	119.9	C7B—C8B—H8BA	119.9
C10A—C9A—C8A	118.3 (2)	C8B—C9B—C10B	117.4 (2)
C10A—C9A—C12A	121.2 (2)	C8B—C9B—C12B	121.1 (2)
C8A—C9A—C12A	120.5 (2)	C10B—C9B—C12B	121.5 (2)
C9A—C10A—C11A	118.0 (2)	C9B—C10B—C11B	118.8 (2)
C9A—C10A—C23A	121.0 (2)	C9B—C10B—C23B	121.1 (2)
C11A—C10A—C23A	121.0 (2)	C11B—C10B—C23B	120.0 (2)
N1A—C11A—O1A	119.7 (2)	N1B—C11B—O1B	121.1 (2)
N1A—C11A—C10A	123.5 (2)	N1B—C11B—C10B	123.4 (2)
O1A—C11A—C10A	116.8 (2)	O1B—C11B—C10B	115.5 (2)
C13A—C12A—C17A	119.3 (2)	C13B—C12B—C17B	119.0 (2)
C13A—C12A—C9A	120.6 (2)	C13B—C12B—C9B	120.9 (2)
C17A—C12A—C9A	120.1 (2)	C17B—C12B—C9B	120.1 (2)
O2A—C13A—C12A	115.6 (2)	O2B—C13B—C14B	123.4 (2)
O2A—C13A—C14A	123.6 (2)	O2B—C13B—C12B	116.5 (2)
C12A—C13A—C14A	120.7 (2)	C14B—C13B—C12B	120.1 (2)
C15A—C14A—C13A	119.6 (2)	C15B—C14B—C13B	120.1 (2)
C15A—C14A—H14A	120.2	C15B—C14B—H14B	120.0
C13A—C14A—H14A	120.2	C13B—C14B—H14B	120.0
O3A—C15A—C14A	123.9 (2)	O3B—C15B—C14B	124.0 (2)
O3A—C15A—C16A	115.8 (2)	O3B—C15B—C16B	115.7 (2)
C14A—C15A—C16A	120.4 (2)	C14B—C15B—C16B	120.3 (2)
O4A—C16A—C17A	124.8 (2)	O4B—C16B—C17B	125.3 (2)
O4A—C16A—C15A	116.0 (2)	O4B—C16B—C15B	115.8 (2)
C17A—C16A—C15A	119.2 (2)	C17B—C16B—C15B	118.9 (2)
C16A—C17A—C12A	120.8 (2)	C16B—C17B—C12B	121.5 (2)
C16A—C17A—H17A	119.6	C16B—C17B—H17B	119.2
C12A—C17A—H17A	119.6	C12B—C17B—H17B	119.2
O1A—C18A—C19A	107.7 (2)	O1B—C18B—C19B	110.5 (2)
O1A—C18A—H18A	110.2	O1B—C18B—H18C	109.6
C19A—C18A—H18A	110.2	C19B—C18B—H18C	109.6
O1A—C18A—H18B	110.2	O1B—C18B—H18D	109.6
C19A—C18A—H18B	110.2	C19B—C18B—H18D	109.6
H18A—C18A—H18B	108.5	H18C—C18B—H18D	108.1
C18A—C19A—H19A	109.5	C18B—C19B—H19D	109.5
C18A—C19A—H19B	109.5	C18B—C19B—H19E	109.5
H19A—C19A—H19B	109.5	H19D—C19B—H19E	109.5
C18A—C19A—H19C	109.5	C18B—C19B—H19F	109.5
H19A—C19A—H19C	109.5	H19D—C19B—H19F	109.5
H19B—C19A—H19C	109.5	H19E—C19B—H19F	109.5
O2A—C20A—H20A	109.5	O2B—C20B—H20D	109.5
O2A—C20A—H20B	109.5	O2B—C20B—H20E	109.5
H20A—C20A—H20B	109.5	H20D—C20B—H20E	109.5
O2A—C20A—H20C	109.5	O2B—C20B—H20F	109.5
H20A—C20A—H20C	109.5	H20D—C20B—H20F	109.5
H20B—C20A—H20C	109.5	H20E—C20B—H20F	109.5
O3A—C21A—H21A	109.5	O3B—C21B—H21D	109.5
O3A—C21A—H21B	109.5	O3B—C21B—H21E	109.5
H21A—C21A—H21B	109.5	H21D—C21B—H21E	109.5
O3A—C21A—H21C	109.5	O3B—C21B—H21F	109.5
H21A—C21A—H21C	109.5	H21D—C21B—H21F	109.5
H21B—C21A—H21C	109.5	H21E—C21B—H21F	109.5
O4A—C22A—H22A	109.5	O4B—C22B—H22D	109.5
O4A—C22A—H22B	109.5	O4B—C22B—H22E	109.5
H22A—C22A—H22B	109.5	H22D—C22B—H22E	109.5
O4A—C22A—H22C	109.5	O4B—C22B—H22F	109.5
H22A—C22A—H22C	109.5	H22D—C22B—H22F	109.5
H22B—C22A—H22C	109.5	H22E—C22B—H22F	109.5
N2A—C23A—C10A	176.9 (3)	N2B—C23B—C10B	177.5 (3)
			
C6A—C1A—C2A—C3A	−0.1 (4)	C6B—C1B—C2B—C3B	0.0 (4)
C1A—C2A—C3A—C4A	−0.7 (4)	C1B—C2B—C3B—C4B	0.4 (4)
C1A—C2A—C3A—Br1A	−179.6 (2)	C1B—C2B—C3B—Br1B	−179.6 (2)
C2A—C3A—C4A—C5A	0.7 (4)	C2B—C3B—C4B—C5B	0.5 (5)
Br1A—C3A—C4A—C5A	179.6 (2)	Br1B—C3B—C4B—C5B	−179.5 (2)
C3A—C4A—C5A—C6A	−0.1 (4)	C3B—C4B—C5B—C6B	−1.8 (5)
C2A—C1A—C6A—C5A	0.7 (4)	C2B—C1B—C6B—C5B	−1.3 (4)
C2A—C1A—C6A—C7A	−180.0 (2)	C2B—C1B—C6B—C7B	176.5 (3)
C4A—C5A—C6A—C1A	−0.6 (4)	C4B—C5B—C6B—C1B	2.2 (4)
C4A—C5A—C6A—C7A	−180.0 (2)	C4B—C5B—C6B—C7B	−175.6 (3)
C11A—N1A—C7A—C8A	0.6 (4)	C11B—N1B—C7B—C8B	3.5 (4)
C11A—N1A—C7A—C6A	−178.3 (2)	C11B—N1B—C7B—C6B	−177.2 (2)
C1A—C6A—C7A—N1A	10.6 (4)	C1B—C6B—C7B—N1B	−27.5 (4)
C5A—C6A—C7A—N1A	−170.1 (2)	C5B—C6B—C7B—N1B	150.2 (3)
C1A—C6A—C7A—C8A	−168.3 (2)	C1B—C6B—C7B—C8B	151.9 (3)
C5A—C6A—C7A—C8A	11.0 (4)	C5B—C6B—C7B—C8B	−30.4 (4)
N1A—C7A—C8A—C9A	−1.8 (4)	N1B—C7B—C8B—C9B	0.1 (4)
C6A—C7A—C8A—C9A	177.1 (2)	C6B—C7B—C8B—C9B	−179.2 (2)
C7A—C8A—C9A—C10A	2.4 (4)	C7B—C8B—C9B—C10B	−3.1 (4)
C7A—C8A—C9A—C12A	−175.0 (2)	C7B—C8B—C9B—C12B	177.3 (2)
C8A—C9A—C10A—C11A	−1.8 (4)	C8B—C9B—C10B—C11B	2.6 (4)
C12A—C9A—C10A—C11A	175.5 (2)	C12B—C9B—C10B—C11B	−177.8 (2)
C8A—C9A—C10A—C23A	177.0 (3)	C8B—C9B—C10B—C23B	−173.4 (3)
C12A—C9A—C10A—C23A	−5.7 (4)	C12B—C9B—C10B—C23B	6.2 (4)
C7A—N1A—C11A—O1A	179.6 (2)	C7B—N1B—C11B—O1B	175.4 (2)
C7A—N1A—C11A—C10A	−0.1 (4)	C7B—N1B—C11B—C10B	−4.0 (4)
C18A—O1A—C11A—N1A	1.7 (3)	C18B—O1B—C11B—N1B	−1.4 (4)
C18A—O1A—C11A—C10A	−178.6 (2)	C18B—O1B—C11B—C10B	178.0 (2)
C9A—C10A—C11A—N1A	0.7 (4)	C9B—C10B—C11B—N1B	1.0 (4)
C23A—C10A—C11A—N1A	−178.1 (3)	C23B—C10B—C11B—N1B	177.1 (2)
C9A—C10A—C11A—O1A	−179.0 (2)	C9B—C10B—C11B—O1B	−178.5 (2)
C23A—C10A—C11A—O1A	2.2 (4)	C23B—C10B—C11B—O1B	−2.4 (4)
C10A—C9A—C12A—C13A	−61.4 (4)	C8B—C9B—C12B—C13B	−114.5 (3)
C8A—C9A—C12A—C13A	115.8 (3)	C10B—C9B—C12B—C13B	66.0 (4)
C10A—C9A—C12A—C17A	117.6 (3)	C8B—C9B—C12B—C17B	64.1 (4)
C8A—C9A—C12A—C17A	−65.2 (4)	C10B—C9B—C12B—C17B	−115.5 (3)
C20A—O2A—C13A—C12A	171.4 (3)	C20B—O2B—C13B—C14B	−5.5 (4)
C20A—O2A—C13A—C14A	−11.6 (4)	C20B—O2B—C13B—C12B	173.9 (2)
C17A—C12A—C13A—O2A	176.7 (2)	C17B—C12B—C13B—O2B	−176.9 (2)
C9A—C12A—C13A—O2A	−4.2 (4)	C9B—C12B—C13B—O2B	1.6 (4)
C17A—C12A—C13A—C14A	−0.3 (4)	C17B—C12B—C13B—C14B	2.5 (4)
C9A—C12A—C13A—C14A	178.7 (2)	C9B—C12B—C13B—C14B	−179.0 (3)
O2A—C13A—C14A—C15A	−177.5 (2)	O2B—C13B—C14B—C15B	179.6 (2)
C12A—C13A—C14A—C15A	−0.7 (4)	C12B—C13B—C14B—C15B	0.3 (4)
C21A—O3A—C15A—C14A	0.2 (4)	C21B—O3B—C15B—C14B	3.8 (4)
C21A—O3A—C15A—C16A	180.0 (2)	C21B—O3B—C15B—C16B	−175.5 (2)
C13A—C14A—C15A—O3A	−179.9 (2)	C13B—C14B—C15B—O3B	178.7 (2)
C13A—C14A—C15A—C16A	0.3 (4)	C13B—C14B—C15B—C16B	−2.1 (4)
C22A—O4A—C16A—C17A	8.1 (4)	C22B—O4B—C16B—C17B	−7.7 (4)
C22A—O4A—C16A—C15A	−172.8 (2)	C22B—O4B—C16B—C15B	173.3 (2)
O3A—C15A—C16A—O4A	2.1 (4)	O3B—C15B—C16B—O4B	−0.6 (3)
C14A—C15A—C16A—O4A	−178.0 (2)	C14B—C15B—C16B—O4B	−179.9 (2)
O3A—C15A—C16A—C17A	−178.7 (2)	O3B—C15B—C16B—C17B	−179.6 (2)
C14A—C15A—C16A—C17A	1.1 (4)	C14B—C15B—C16B—C17B	1.1 (4)
O4A—C16A—C17A—C12A	176.9 (2)	O4B—C16B—C17B—C12B	−177.2 (2)
C15A—C16A—C17A—C12A	−2.1 (4)	C15B—C16B—C17B—C12B	1.7 (4)
C13A—C12A—C17A—C16A	1.8 (4)	C13B—C12B—C17B—C16B	−3.5 (4)
C9A—C12A—C17A—C16A	−177.3 (2)	C9B—C12B—C17B—C16B	177.9 (2)
C11A—O1A—C18A—C19A	−174.0 (2)	C11B—O1B—C18B—C19B	92.8 (3)

### Hydrogen-bond geometry (Å, °)

**Table:**

Cg1 and Cg2 are the centroids of C7A–C11A/N1A and C12A–C17A rings, respectively.

**Table:**

*D*—H···*A*	*D*—H	H···*A*	*D*···*A*	*D*—H···*A*
C1A—H1AA···N1A	0.93	2.43	2.770 (3)	102
C18B—H18C···N1B	0.97	2.38	2.741 (4)	101
C22A—H22C···Cg2^i^	0.96	2.74	3.585 (3)	147
C22B—H22E···Cg1^ii^	0.96	2.68	3.511 (3)	145

Symmetry codes:  (i) −*x*+1, −*y*+1, −*z*+1; (ii) −*x*, −*y*+1, −*z*+1.
